# Transforming Pediatric Physiotherapy: The Role of ChatGPT in Therapy, Limitations, and Ethical Considerations

**DOI:** 10.7759/cureus.91571

**Published:** 2025-09-03

**Authors:** Hussein M Ziab, Rami Mazbouh, Fatima Siblini, Abdulqadir J Nashwan

**Affiliations:** 1 Al-Maha Pediatric Specialized Care Center, Hamad Medical Corporation, Doha, QAT; 2 Faculty of Public Health, Lebanese University, Beirut, LBN; 3 Department of Physiotherapy, Islamic University of Lebanon, Baalbek, LBN; 4 Department of Research and Development/Physiotherapy, Health, Rehabilitation, Integration and Research Center, Beirut, LBN; 5 School of Rehabilitation, Tehran University of Medical Sciences, Tehran, IRN; 6 Department of Nursing Education and Research, Hamad Medical Corporation, Doha, QAT

**Keywords:** ai ethics, chatgpt, clinical decision support, pediatric rehabilitation, physiotherapy

## Abstract

The use of large language models (LLMs), such as ChatGPT in pediatric physiotherapy, is a big step forward in the provision of tailored care, clinical decision-making, and caregiver education. This narrative review examines ChatGPT's roles in improving physiotherapy practice across three major domains: (1) clinical reasoning, (2) tailored therapeutic recommendations, and (3) caregiver engagement. Drawing on recent empirical and theoretical literature, the paper discusses ChatGPT's ability to generate context-aware, developmentally appropriate content, its limits in transparency, factual correctness, and relational interactivity. Key ethical challenges are examined, including algorithmic bias, data privacy, medico-legal responsibility, and the risk of diminishing humanistic aspects of care. While ChatGPT holds promise as a supplementary tool to support evidence-based rehabilitation, particularly in under-resourced or home-based settings, its effective implementation requires ongoing human oversight, robust validation, and clinician training. The paper concludes with practical recommendations and ethical safeguards for responsible integration, emphasizing that ChatGPT should augment, not replace, human clinical expertise in pediatric rehabilitation contexts.

## Introduction and background

Pediatric physiotherapy demands individualized, developmentally appropriate approaches that address the unique physical and cognitive needs of children. With the growing incorporation of artificial intelligence (AI) technologies into healthcare, their application requires a nuanced understanding of benefits, risks, and ethical implications [1]. Significant advancements in diagnostics, therapeutics, and safety were highlighted in a recent SWOT (strengths, weaknesses, opportunities, and threats) analysis of AI in clinical medicine. However, it also brought to light enduring ethical concerns about bias, privacy, and opacity, which call for strong governance and ongoing clinical oversight [2]. Clinical reliability in pediatric settings is directly threatened by issues with sample variability, where the same prompts may produce inconsistent results, and hallucinations, where the confident presentation of false or erroneous information occurs. These hazards are especially significant in pediatric physical therapy, where safety and efficacy must be guaranteed by developmentally appropriate and evidence-based recommendations.

AI refers to the development of computational systems capable of mimicking human-like intelligence, including learning, reasoning, decision-making, and natural language understanding. Key subfields of AI relevant to healthcare include machine learning, computer vision, robotics, and natural language processing (NLP), the latter of which underpins the functionality of generative language models such as ChatGPT. These tools have already begun to reshape domains such as diagnostics, protocol development, patient education, and telemedicine, with growing interest in their applicability to rehabilitation sciences, including physiotherapy.

One of the most prominent AI tools in healthcare is the generative pre-trained transformer (GPT), which is based on the Transformer architecture introduced in the landmark paper ‘Attention is All You Need’ by Vaswani et al. [3]. The core idea behind the transformer is the use of self-attention mechanisms that process words in relation to all other words in a sentence, contrary to traditional methods that process words in sequential order. ChatGPT, as the largest publicly available language model, is a complex NLP model based on the Transformer architecture, which enables it to understand and generate human-like text by modeling long-range dependencies in language [4,5].

Developed by OpenAI and released on November 30, 2022, ChatGPT was trained on a large and diverse dataset comprising books, articles, and web content, enabling it to generalize across a wide range of topics. The model undergoes a two-phase training process: pre-training on vast text corpora and fine-tuning with human feedback to improve alignment and conversational quality [4]. It tokenizes input using byte-pair encoding (BPE), which helps manage rare and compound words efficiently [6]. ChatGPT maintains dialogue history within a context window, enabling it to generate coherent multi-turn conversations [4]. Responses are generated using probabilistic sampling techniques, such as top-k and nucleus sampling, with temperature control to adjust creativity and determinism [7]. These mechanisms allow ChatGPT to interpret complex prompts, including those related to physiotherapy - such as diagnostic criteria, therapeutic interventions, and patient education - and provide context-aware, accurate, and professionally relevant information [4].

Within the clinical decision-support systems (CDSS) framework, decision-making in physiotherapy can be conceptualized as four sequential stages: (1) data collection, (2) clinical reasoning in diagnosis, (3) intervention planning, and (4) progress monitoring [8]. This framework has been widely applied in rehabilitation to structure clinical reasoning, ensuring that assessments, diagnoses, and interventions are systematically aligned with patient-specific needs [9]. In pediatric physiotherapy, these stages also demand integration of developmental milestones, individualized family goals, and multidisciplinary collaboration elements that introduce complexities not typically present in adult care. Traditional pediatric physiotherapy workflows are clinician-led at every stage, relying on structured assessments, standardized outcome measures, family interviews, and iterative adjustments informed by hands-on observation. These processes are grounded in tacit clinical knowledge, contextual understanding, and rapport-building with children and caregivers.

Other AI tools - such as robotics, computer vision, or rule-based expert systems - tend to focus on specific, structured tasks (e.g., delivering therapy, quantifying movement, or classifying outcomes). In contrast, ChatGPT’s strength lies in language-based reasoning and its capacity to synthesize information across unstructured inputs, positioning it to support stages 1-3 of the CDSS model [10].

From a human-AI teaming perspective, empirical findings suggest that tenders of effective collaboration in clinical settings depend on integrating AI as a complementary knowledge source, not a replacement. Studies have shown that AI can enhance hypothesis generation and speaker engagement when integrated properly into team workflows [11].

Moreover, ChatGPT’s outputs are probabilistic, meaning that repeat queries may yield different responses, and hallucinations - producing confident but incorrect answers - are known risks. Consequently, human oversight remains essential. Adoption will depend not only on capability but also on human factors-trust, perceived utility, and ease of use - as emphasized in technology acceptance literature (e.g., technology acceptance model (TAM), unified theory of acceptance and use of technology (UTAUT)). One integrative review applying the UTAUT model to AI adoption in hospitals highlights barriers such as fear of loss of autonomy and difficulty in workflow integration as key inhibitors to acceptance [12].

In physiotherapy, AI tools such as ChatGPT offer promising avenues to enhance clinical reasoning, improve diagnostic precision, streamline treatment planning, and support protocol development and customization by reducing time and increasing accuracy. Recent evidence has shown that ChatGPT-4 aligns with clinical practice guidelines in 80% of musculoskeletal care scenarios, with excellent inter-rater reliability (κ = 0.847), highlighting its potential as a supplementary decision-support tool in clinical practices [13].

Complementing this, qualitative findings from physiotherapy education revealed that students perceived generative AI as a valuable learning partner - supporting clinical reasoning, problem-solving, and efficient assimilation of complex course material - while emphasizing the importance of critical thinking and institutional guidance to mitigate risks of over-reliance and factual inaccuracies [14]. Similarly, recent evidence from pediatric rehabilitation research further highlights the transformative potential of AI tools in clinical settings. A scoping review by Kaelin et al. systematically examined the application of AI in pediatric rehabilitation, with a focus on interventions aimed at enhancing children's participation. However, the review highlighted critical limitations, including a predominant focus on in-person interventions, minimal integration of individualized goal-setting, and limited personalization based on child- or family-reported participation needs [15].

Within this context, ChatGPT has shown potential in enhancing efficiency and reducing clinician burden by supporting clinical decision-making through its ability to interpret queries and provide recommendations. However, its role in enhancing patient-centered care - such as facilitating shared decision-making, goal setting, and personalized education - as well as the accuracy of its answers, remains largely theoretical and is not directly supported by current empirical studies in pediatric physiotherapy. For instance, Andykarayalar et al. evaluated ChatGPT’s performance in pediatric case scenarios and found that, while it could offer comprehensive information in some cases, it also demonstrated notable inaccuracies in others. Their findings underscore the importance of cautious integration and the need for expert validation, particularly in sensitive pediatric contexts [16]. Therefore, while ChatGPT may contribute to more informed interactions, its capacity to support truly person-centered care requires further investigation and validation.

With growing but fragmented literature, a critical, pediatrics-focused synthesis is essential. The narrative review, then, attempts to outline ChatGPT's potential applications, benefits, limitations, ethical implications, and future directions in pediatric physiotherapy clinical practice, namely, refraining from undue generalizations from other non-pediatric applications and filling evidence gaps.

## Review

Methods

This narrative review did not follow a standardized systematic search strategy; however, literature was identified through targeted searches in PubMed, Scopus, and Google Scholar. The search focused on studies published between 2010 and July 2024 using key terms, such as “artificial intelligence”, “ChatGPT”, “pediatric rehabilitation”, “physiotherapy”, and “clinical decision support”. Additional references were retrieved through manual screening of bibliographies from relevant articles. The timeframe was chosen to capture both early developments in healthcare-related AI (post-2010) and the most recent literature following the release of ChatGPT in 2022. Moreover, no restrictions were applied regarding study design, quality, or population characteristics, as the primary aim was to clarify the potential role of ChatGPT in physiotherapy, with a particular focus on pediatric contexts.

Applications for pediatric physiotherapy

Clinical Decision-Making Support

ChatGPT can assist clinicians by synthesizing existing evidence, generating summaries of treatment modalities, and identifying patterns across large datasets - thereby supporting clinical reasoning, especially in pediatric cases involving complex rehabilitation needs. Its ability to simulate decision pathways can help physiotherapists refine parameters such as therapy frequency, exercise progression, and discharge planning by referencing similar clinical scenarios [17].

Recent comparative evaluations have further highlighted the capabilities of advanced AI models such as ChatGPT o1, which achieved a diagnostic accuracy of 92.8% when tested on 500 pediatric clinical questions from the MedQA dataset [18]. This performance was significantly higher than that of other models and was attributed to its use of chain-of-thought reasoning, which enables structured, stepwise problem-solving. Such capacity renders ChatGPT o1 particularly well-suited for pediatric clinical decision-making, where nuanced and error-sensitive judgments are required.

However, findings from Andykarayalar et al. [16] underscore the variability in ChatGPT’s performance across pediatric subspecialties. In their evaluation of ChatGPT-3.5 using 24 clinical scenarios from “100 Cases in Pediatrics”, the model demonstrated inconsistent diagnostic accuracy, with several cases graded as “C” for incorrect responses - particularly in critical conditions such as congenital heart disease, septic shock, and acute pyelonephritis. These results highlight the importance of cautious integration and expert oversight when using ChatGPT as a clinical decision support system (CDSS) in pediatric care. While the model can provide comprehensive information in some scenarios, it is not yet reliable enough to replace clinical judgment and should be used as a supplementary tool, especially in complex or high-risk cases [16].

These discrepancies likely stem from differences in model architecture, reasoning strategies (chain-of-thought vs. single-pass generation), and the complexity of the cases presented. Collectively, the evidence suggests that ChatGPT’s reliability is the highest in structured, knowledge-rich conditions with clear diagnostic pathways and the weakest in rare, multi-system, or acute pediatric presentations. For pediatric physiotherapy, this implies that current models may be better suited to supporting decision-making in predictable rehabilitation scenarios (e.g., post-orthopedic protocols) than in high-acuity triage or differential diagnosis. Future research should focus on developing and validating pediatric physiotherapy-specific benchmarking datasets and conducting prospective trials comparing AI-assisted and clinician-only decision-making.

Tailored Exercise Recommendations

ChatGPT, when paired with adaptive prompting and clinical oversight, shows considerable potential in supporting physiotherapists by suggesting developmentally appropriate exercises and individualized therapeutic strategies. While it does not replace professional judgment or prescribe definitive rehabilitation protocols, ChatGPT provides scaffolding prompts that therapists can critically appraise and adapt to meet developmental and therapeutic needs. Twenty standardized questions covering three domains - disease information, patient assessment, and rehabilitation - distributed throughout the upper extremities, lower extremities, and spine were used in a cross-sectional observational study in musculoskeletal physiotherapy by Safran et al. [13]. Two expert physiotherapists independently assessed ChatGPT's responses for completeness, accuracy, consistency, clarity, and relevancy. The model performed best in illness information, achieving up to 80% alignment with clinical practice standards; however, its outputs in rehabilitation showed poorer inter-rater agreement and more variability [13]. This highlights its utility as a supplementary - rather than primary - decision-support tool, particularly in less complex or standardized therapy scenarios. Similarly, in the context of pediatric physical activity promotion, Willms et al. [19] demonstrated that ChatGPT could be leveraged to generate structured, personalized content for mobile health interventions using the multi-process action control (M-PAC) framework. Their study showed that ChatGPT could effectively produce tailored behavioral content through a six-step process that included defining the target behavior, grounding the intervention in behavior change theory, and refining responses for use in just-in-time adaptive interventions (JITAIs). Although professional oversight remains essential to ensure clinical accuracy, this work underscores ChatGPT’s potential in supporting tailored, theory-driven interventions for pediatric populations [19].

For pediatric rehabilitation, this capability offers a promising avenue to bridge service gaps - especially in under-resourced or home-based care settings. The model’s responsiveness to user inputs can support continuity of care, reinforce caregiver adherence, and enhance engagement in daily routines. As digital health increasingly prioritizes accessible and tailored interventions, the integration of AI tools such as ChatGPT may offer an innovative, scalable solution to support individualized and evidence-aligned rehabilitation strategies for children.

Enhancing Caregiver Engagement and Education

Parents and caregivers often face challenges in understanding and adhering to complex pediatric physiotherapy protocols, particularly when confronted with medical jargon, variable communication styles, or limited health literacy. It has been hypothesized that ChatGPT, with its advanced natural language processing capabilities, could offer a flexible and accessible tool by adapting explanations to diverse cognitive levels, literacy skills, and language backgrounds; however, this potential remains to be empirically validated in clinical settings [20].

Notably, ChatGPT can produce responses that simulate the tone of what it refers to as “a friendly and intelligent robot.” Its core strengths include the ability to respond to complex and open-ended questions, manage multi-turn conversations, and maintain coherence and consistency throughout the interaction.

Research highlights that large language models (LLMs) such as ChatGPT can simplify technical language, use analogies and metaphors, and adjust tone to suit lay audiences - features that are critical for caregiver comprehension and engagement, and health communication efficacy [17]. For instance, it may explain "range of motion" as "how far your child can move their arm, like swinging it forward or reaching behind," using metaphors or child-friendly analogies to make abstract clinical terms more relatable (refer to Table 1).

**Table 1:** Sample of examples on how ChatGPT can support caregiver engagement This table provides examples of how ChatGPT answers some queries parents may request. It was originally designed by the authors. The educational function represents the benefits the authors consider from using ChatGPT.

Caregiver Prompt	ChatGPT Response	Educational Function
“My child has cerebral palsy. What kind of exercises can help her walk better?”	“I can suggest some gentle stretching and strengthening exercises that can support walking. For example, calf stretches and sit-to-stand practice help improve leg strength. However, it’s important to follow your physiotherapist’s advice for a program that fits your child’s needs”.	Provides accessible guidance; encourages collaboration with clinician
“He doesn’t like doing exercises at home. What can I do to motivate him?”	“You can try turning the exercises into a game. For example, you can say, ‘Let’s see how far you can reach like a superhero!’ Giving praise and using music or rewards can also make therapy fun”.	Offers behavior management and motivational strategies
“What does ‘gross motor delay’ mean?”	“It means your child is taking longer to learn big movements like crawling, standing, or jumping. Every child is different, but with support, they can keep improving at their own pace”.	Simplifies medical jargon through analogy and reassurance
“Is it okay if we miss some sessions due to travel?”	“It’s best to follow the therapy schedule, but if you’re traveling, your therapist may give you home activities. Staying consistent helps your child progress”.	Supports adherence and empowers proactive planning
“Can you explain what ‘range of motion’ means?”	“It’s how far your child can move a part of their body—like swinging their arm up or turning their head to the side. We want to keep these movements smooth and comfortable”.	Clarifies clinical terms using child-friendly language and metaphors

This linguistic adaptability may enhance the therapeutic alliance by enabling more inclusive and collaborative communication. Emerging research demonstrates that ChatGPT can provide real-time, emotionally supportive responses to caregivers, with potential for cultural adaptability and contextual relevance, particularly when queries are specific [21]. These interactions support shared decision-making and build caregiver confidence-factors associated with improved adherence to home exercise programs and better outcomes in pediatric rehabilitation [15,17].

Nevertheless, despite its promise, the application of ChatGPT must be supported by clinician oversight to ensure contextual appropriateness and evidence-based accuracy. When properly integrated, ChatGPT functions as a valuable supplementary tool-enhancing caregiver understanding, especially in under-resourced or telehealth settings where direct therapist contact may be limited. Empirical trials are needed to determine whether AI-mediated education improves caregiver adherence, confidence, or child functional outcomes, particularly in culturally and linguistically diverse populations.

Limitations of ChatGPT in pediatric physiotherapy

Despite its expanding applications, the integration of ChatGPT into pediatric physiotherapy presents multiple limitations that require careful consideration. One of the most pressing concerns is the risk of factual inaccuracies, especially when ChatGPT generates responses based on incomplete or ambiguous prompts. The phenomenon of "hallucinations,” wherein the AI produces authoritative-sounding but fabricated or incorrect information or references, is well-documented and presents significant risks in clinical and academic contexts [2,22]. This issue is especially problematic in pediatric care, where nuanced and context-specific information is vital. For example, Andykarayalar et al. [16] found that, while ChatGPT could offer comprehensive responses to some pediatric case scenarios, it demonstrated critical inaccuracies in others, particularly in conditions such as congenital heart disease, septic shock, and acute pyelonephritis [16]. These findings underscore the need for expert oversight and cautious integration into clinical practice.

In relation to this, much of the existing evidence base, though, originates from adult musculoskeletal or general rehabilitation, and results have routinely been overgeneralized from these contexts to pediatric physiotherapy without empirical verification. Such overgeneralizations of claims have the potential to mask the distinct developmental, physiological, and family-oriented considerations that are part and parcel of pediatric practice (Table 2). The necessity, then, is a critical examination of ChatGPT’s application within pediatric-specific contexts, rather than presuming equivalency across populations.

**Table 2:** Traditional vs ChatGPT-augmented pediatric physiotherapy within the CDSS framework This table was created originally by the authors. It summarizes the findings from the literature. CDSS: clinical decision-support systems

CDSS Stage	Traditional Pediatric Physiotherapy	ChatGPT-Augmented Approach
Data collection	The therapist collects history, conducts physical examinations, consults caregiver reports, and reviews medical records.	ChatGPT synthesizes caregiver narratives, clinical notes, and guidelines into structured summaries and prompts for missing information.
Diagnostic reasoning	The clinician applies developmental norms, standardized tests, and experience to identify issues.	ChatGPT suggests possible differential diagnoses, relevant tests, and evidence-based references, highlighting guideline-aligned options.
Intervention	The therapist designs a protocol based on guidelines, patient goals, and constraints (e.g., session frequency, environment).	ChatGPT proposes intervention options, dosage parameters, and progression criteria based on input variables.
Progress monitoring	The clinician monitors progress using outcome measures and adjusts the plan accordingly.	ChatGPT summarizes progress notes, identifies trends, and suggests possible adjustments (pending clinician validation).

Additionally, ChatGPT’s outputs can lack transparency due to the “black box” nature of deep learning, making it difficult for clinicians to understand the rationale behind specific recommendations [23,24]. This opacity limits its utility in shared decision-making and informed consent processes, which are foundational to ethical pediatric care. Concerns have also been raised about authorship and accountability when AI-generated content is used in clinical documentation or scientific communication, particularly given the model’s capacity to fabricate references or cite outdated evidence [14,25]. Furthermore, the influence of commercial training data and the absence of transparency regarding data provenance raise the risk of knowledge gaps or bias, especially in underrepresented pediatric populations.

A further limitation stems from ChatGPT’s inability to assess nonverbal, sensory, and neuromotor cues, which are fundamental to pediatric physiotherapy evaluation. The model lacks real-time perceptual capabilities, such as observing movement patterns, interpreting play behavior, or assessing postural control - skills that are central to tailoring therapeutic interventions in children [15]. As such, any recommendations it offers must be critically reviewed within the framework of a full clinical assessment.

Moreover, as of its latest public release, ChatGPT is trained on data available only up to April 2023 and does not have access to proprietary or discipline-specific clinical databases [4]. These temporal and epistemic limitations constrain its ability to reflect the most current research in pediatric rehabilitation or the latest updates in clinical guidelines. Additionally, the model is trained predominantly on English-language and high-income country data, which raises concerns about cultural bias and transparency - particularly in low-resource or cross-cultural contexts where AI-generated recommendations may not align with localized practices or culturally responsive care [21,26].

Finally, there are humanistic and relational limitations. Pediatric physiotherapy depends heavily on empathy, trust-building, and playful interaction - elements that cannot be authentically replicated by text-based AI. Overreliance on ChatGPT may unintentionally diminish these relational aspects, weakening the therapeutic alliance and caregiver engagement, particularly when used without contextual sensitivity or personal communication [17].

Taken together, these limitations emphasize that ChatGPT should be used only as a supplementary decision-support and educational tool, never as a substitute for clinical reasoning, hands-on assessment, or interpersonal care. Its safe and effective use in pediatric physiotherapy requires strict human oversight, ongoing model validation, and integration within ethical and evidence-based practice frameworks.

Practical recommendations for clinicians

Institutions and professional bodies should begin embedding foundational AI literacy into physiotherapy training programs. According to Naqvi et al. [17], this includes equipping clinicians with the skills to understand algorithmic bias, structure effective prompts, and evaluate AI outputs, thereby fostering a generation of physiotherapists capable of leading responsible digital innovation in care delivery [17].

**Figure 1:**
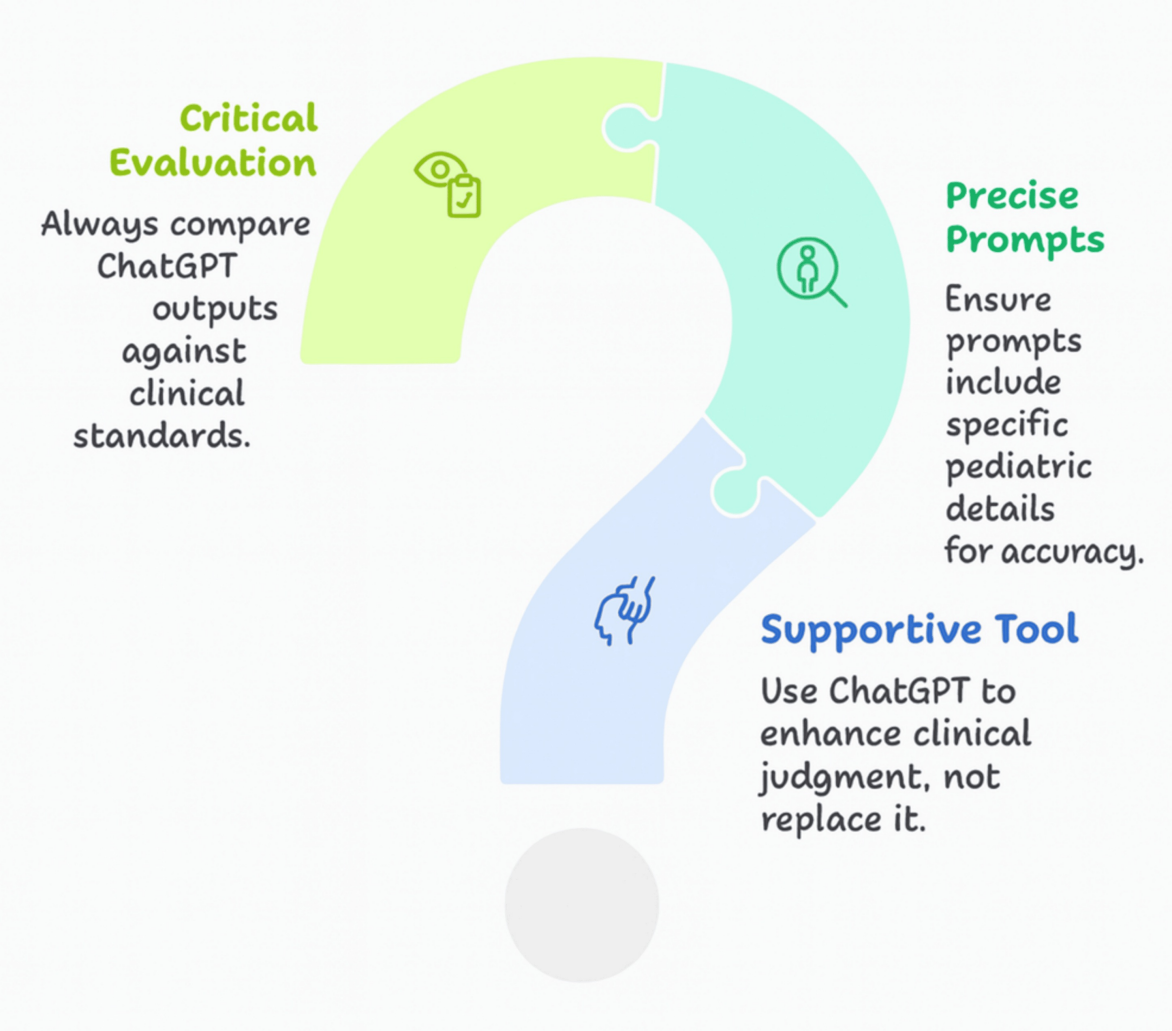
Integration of ChatGPT in pediatric physiotherapy daily practice This figure was built using the Napkin AI tool. It summarizes the text. It was designed originally by the authors.

The integration of ChatGPT into pediatric physiotherapy not only presents significant opportunities but also necessitates a cautious, ethically grounded approach (Figure 1). From a practical standpoint, clinicians should ensure that ChatGPT is employed as a supportive tool rather than a replacement for clinical judgment. This involves using precise, context-specific prompts that include relevant pediatric details, such as developmental stage, diagnosis, and therapy goals, to enhance response accuracy and relevance [14]. Given ChatGPT’s limitations in domain-specific reasoning, outputs should always be critically evaluated against evidence-based standards and current clinical guidelines.

To ensure clinically relevant AI integration, physiotherapists must be empowered not just as end-users but as co-developers of AI systems. More practical advancements could be made by interdisciplinary collaboration with data scientists, where PTs contribute domain expertise while acquiring fundamental knowledge in areas such as prompt engineering and data curation, as opposed to mandating that PTs undergo intensive coding training. The creation of evidence-based, ethically educated, and therapeutically responsive LLMs tailored to physiotherapy might be aided by such collaboration as proposed by Naqvi et al. [17].

Ethical considerations for ChatGPT use in pediatric physiotherapy

The integration of ChatGPT into pediatric physiotherapy practice offers transformative potential for clinical reasoning, caregiver education, and therapeutic engagement. However, such integration must be guided by rigorous ethical safeguards, particularly given the vulnerable nature of pediatric populations and the complexities of child development (refer to Table 3).

**Table 3:** Ethical risks of ChatGPT in pediatric physiotherapy and suggested mitigation strategies This is an original work designed by the authors to summarize their findings. It correlates the ethical risks with the mitigation strategies that are already cited in the text.

Ethical Risk	Description	Mitigation Strategy
Factual Inaccuracy (“Hallucinations”)	ChatGPT may generate plausible but incorrect or misleading information.	Implement layered human validation, cross-check outputs with clinical guidelines, and restrict use to low-risk contexts.
Opacity/Lack of Transparency	Users cannot easily trace how ChatGPT generated a specific response or on what data it was based.	Encourage the use of explainable AI systems, inform users about model limitations, and avoid sole reliance for decision-making.
Data Privacy & Confidentiality Breach	Public ChatGPT lacks HIPAA compliance and encryption; patient data could be exposed.	Avoid sharing identifiable patient data; use anonymized cases; ensure institutional policies and informed disclosure.
Algorithmic Bias	ChatGPT is trained mostly on English-language, high-income datasets, risking biased outputs.	Promote regular audits, use culturally adapted prompts, and involve clinicians in verifying equity in outputs.
Erosion of Therapeutic Relationship	Overuse may weaken human empathy, trust, and the clinician-caregiver bond.	Use ChatGPT only to supplement—not replace—interpersonal communication; preserve time for direct therapist-caregiver interaction.
Lack of Regulatory Accountability	ChatGPT is not a licensed medical device; no legal liability for inaccurate outputs.	Clarify clinician responsibility, ensure outputs are advisory, and advocate for future legal frameworks.
Informed Consent Challenges	Patients/caregivers may be unaware of AI’s role in care or communication.	Disclose AI use clearly, obtain consent, and include AI in caregiver education discussions.

Legal and Professional Responsibility

Healthcare professionals retain full legal and ethical accountability for clinical decisions informed by ChatGPT. As a general-purpose language model, ChatGPT is not a certified medical device and cannot assume regulatory liability. Its outputs must be interpreted as advisory - not prescriptive - requiring healthcare providers to maintain ultimate authority and apply clinical judgment in all decisions [9]. Clear legal frameworks and institutional governance are necessary to delineate the responsibilities of clinicians, AI developers, and healthcare systems - ensuring accountability, fairness, and trust in AI integration protocols [27,28].

Information Accuracy and Human Oversight

A central ethical concern is the risk of misinformation, particularly through "hallucinations," wherein ChatGPT generates plausible yet factually incorrect content. In pediatric care, where nuanced developmental considerations and caregiver perceptions influence outcomes, such inaccuracies can have amplified consequences. Therefore, AI outputs must undergo thorough validation by clinicians before informing practice [26]. The ethics debate should shift toward defining context-sensitive thresholds of human oversight-differentiated by user expertise, risk profile, and criticality of the clinical application.

As Naqvi et al. [17] noted, hallucinations - AI-generated content that appears plausible but is factually incorrect - are a central threat to clinical safety. They recommend layered review systems involving expert validation and benchmarking against gold-standard datasets. Applying such safeguards in pediatric physiotherapy would help mitigate diagnostic or instructional inaccuracies, particularly in high-stakes rehabilitation planning.

Patient Privacy and Data Protection

Data ethics refers to how you collect, store, and use the data of your patients. Interactions with ChatGPT should never include personally identifiable health information. The absence of encryption or institutional controls in publicly available LLMs exposes users to significant data security risks. Clinicians must ensure compliance with privacy laws such as the Health Insurance Portability and Accountability Act (HIPAA), use anonymized input formats, and disclose AI use to caregivers to maintain transparency and trust [29-31]. It is crucial to ensure that patient data are utilized solely for the purpose of enhancing care and improving patient outcomes.

Humanistic and Relational Ethics

The therapeutic alliance in pediatric physiotherapy relies on empathy, trust, and human connection. Overreliance on AI tools risks undermining this dynamic, particularly if ChatGPT is used in place of direct clinician communication. Its role should be explicitly defined as augmenting - rather than replacing - the human elements of care. Practitioners must ensure that their deployment supports shared decision-making and reinforces, rather than diminishes, caregiver engagement [10].

Algorithmic Bias and Fairness

ChatGPT is trained on datasets that disproportionately represent knowledge from high-income, English-speaking contexts. This poses ethical risks of cultural insensitivity, diagnostic bias, and inequitable treatment recommendations [30]. In pediatric rehabilitation, where culturally responsive and individualized care is paramount, such biases may exacerbate disparities. Regular auditing, interpretability mechanisms, and clinician awareness are essential to ensure equity in AI-supported care [10,26].

Transparency and Informed Consent

Given the inherent opacity of LLMs, especially regarding the provenance of generated content, clinicians must take proactive steps to maintain transparency. This includes informing caregivers when AI tools are used in therapeutic planning or education and obtaining explicit consent where appropriate. Such transparency helps mitigate erosion of trust and reinforces ethical standards of informed participation in care [26].

## Conclusions

ChatGPT holds considerable potential as an adjunct tool in pediatric physiotherapy, supporting clinical reasoning, family engagement, and therapy personalization. However, its integration must be approached with caution, respecting ethical boundaries and professional judgment. A balanced strategy that combines AI innovation with human empathy and clinical expertise is key to optimizing outcomes in pediatric rehabilitation.

Future developments in ChatGPT for pediatric physiotherapy should focus on enhancing interactivity through child-friendly interfaces, including visual aids and voice-based engagement, to improve therapeutic participation. Rather than replacing clinicians, ChatGPT should function as a collaborative tool that supports dynamic, therapist-guided interventions and personalized goal setting. To ensure responsible integration, training programs must equip physiotherapists with skills in ethical AI use, data privacy, and critical evaluation of AI-generated content, while multidisciplinary efforts should guide the creation of governance frameworks aligned with clinical standards.
